# Resection of Bilateral Symmetrical Multiple Level Cervical Ganglioneuroma in a 43-Year-Old Man, a Probable Case of Neurofibromatosis Type-1: Report of a Case and Review of Literature

**DOI:** 10.1155/2022/4547572

**Published:** 2022-07-15

**Authors:** Seyed Reza Mousavi, Mohammadhadi Amirshahpari Motlagh, Fatemeh Karimi, Bahareh Ebrahimi

**Affiliations:** ^1^Shiraz Neuroscience Research Center, Shiraz University of Medical Sciences, Shiraz, Iran; ^2^Department of Neurosurgery, Shiraz University of Medical Sciences, Shiraz, Iran; ^3^Histomorphometry and Stereology Research Center, Shiraz University of Medical Sciences, Shiraz, Iran; ^4^Anatomy Department, School of Medicine, Shiraz University of Medical Sciences, Shiraz, Iran; ^5^Shiraz Geriatric Research Center, Shiraz University of Medical Sciences, Shiraz, Iran

## Abstract

Ganglioneuroma is a benign tumor, originating from sympathetic nervous system. Intradural and dumbbell shape spinal ganglioneuroma has been reported in the literature. In this study, we intend to present our case, a 43-year-old man with multiple cutaneous dimples—probably a Neurofibromatosis type-1 (NF-1) case—and subacute myelopathy, who presented with bilateral symmetric dumbbell shape C2/C3 and C4/C5 intradural extramedullary tumors. After resection, the pathologic feature was revealed as ganglioneuroma. We also reviewed the literature for similar cases, which revealed our case to be the 9th bilateral and symmetrical spinal GN, all of which in cervical region; the 5th involving multiple level (the 3rd multiple bilateral symmetrical involvement), the 3rd extending intradurally, and the first case of involving all cervical nerve root ganglions in different sizes. Bilateral symmetrical spinal GNs have also appeared to have different body location, geographic, and gender distribution.

## 1. Introduction

Ganglioneuromas (GNs) are rare benign tumors of the sympathic nervous system with neural crest cell origin that usually are diagnosed in women and in the age below 20 [1–3]. As to the sites, GN is found more commonly in the posterior mediastinum, retroperitoneal space, adrenal medulla, and neck [1]. In this regard, cases of cervical spine GNs presenting with radiculopathy and/or myelopathy are quite rare [3, 4]. Literature review indicates that there is an association between this tumor, multiple endocrine neoplasms (MEN) [5, 6], and Von Recklinghausen's disease (NF-1) [2–4]. Although numerous bilateral symmetrical single-level cervical spine GNs have been reported [2–4, 7–12], multiple level cervical symmetrical dumbbell GNs have been only reported only twice in literatures yet [8, 11]. Our present case is a probable case of Von Recklinghausen's disease (NF-1), with numerous skin dimples since childhood, who presented with cervical pain and myelopathy signs and symptoms.

## 2. Case Presentation

**Figure 1:**
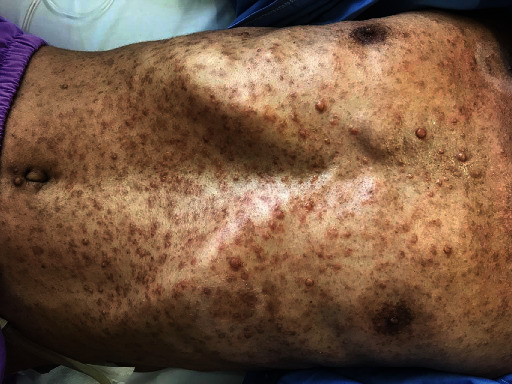
Diffuse cutaneous nodules and café-au-lait spots.

**Figure 2:**
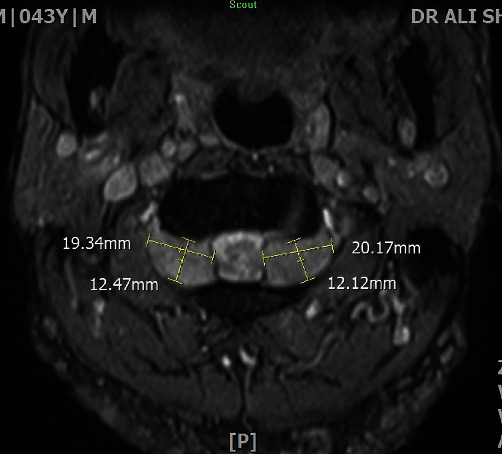
Bilateral symmetrical C2 GN- axial T2WI at C1-C2.

**Figure 3:**
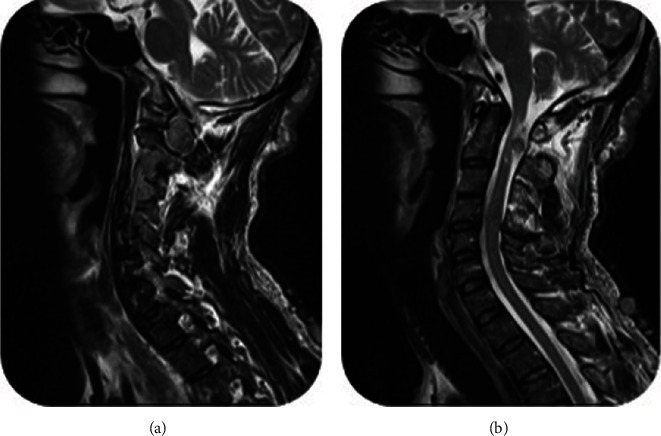
Sagittal T2WI showing expanded intervertebral foramina mostly at C1-C2 level (a) and dorsal intradural part of bilateral C2 ganlion GN, compressing spinal cord and causing signal change at highest cervical spinal cord segments.

**Figure 4:**
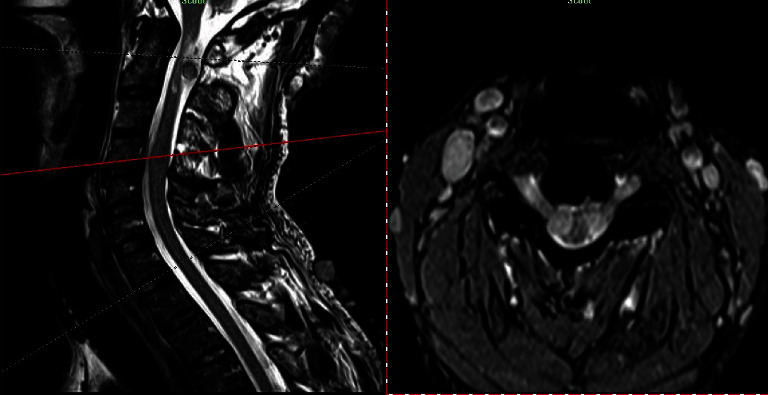
Sagittal-axial T2WI cross view of C4-C5 level showing bilateral symmetrical intradural ventrolateral C4 GN, compressing spinal cord.

Our present case is a 43-year-old constructive worker, who presented with chief complaint of slowly progressive claudication. Having fallen on his right side while walking, he has suffered bilateral upper extremity distal paresis for 2 years, which has worsened since last 2 months and was interfering with his daily working activities. He has also been feeling paresthesia and numbness in his right side. He has had no medical history except an appendectomy. As to drug history, he has been opium dependent for some years. In general, the patient is a young slim man with numerous skin dimples (Figure 1) ranging from a few millimeters to about 2-3 cm diffused all over his skin. Regarding mobility, he was able to walk if receiving little help. In neurologic examination, we found vibration sensory deficit in his lower extremities (Rt > Lt) and spastic gate. He had bilateral distal upper extremity paresis (4/5) and increased deep tendon reflexes (3/4) with bilateral positive Hoffmann's test and Babinski sign. He had no further sensory or sphincter complaint or deficit. After normal routine laboratory tests, brain, and spinal magnetic resonance imaging (MRI) was performed, revealing further findings (Figures 2–4) as follows:

- Normal brain and thoracic spine MRI
- Cervical spine MRI (Figures 2–4)

Multiplanar multisequential MRI images through cervical spine, without contrast injection, revealed multiple subcutaneous lesions that the largest was 10 mm. Evidence of multiple lesions on the right and left bilateral exiting nerve roots were also seen. The most prominent one was at the level of C1-C2 measuring 25 × 16 and 22 × 13 mm in the left and right sides, respectively. The mentioned extra axial lesions extend to the posterior aspect of the cervical thecal sac that causes pressure effect over the cervical cord with increase in signal intensity of the cervical cord at this level. Also, multiple mass lesions involving the exiting nerve roots at the level of C2-C3, C3-C4, C4-C5, C5-C6, C6-C7, and C7-T1 (the visualized levels) were noted; thus, possibility of multiple neuroma or schwannoma should be taken into consideration. Further investigation is recommended to rule out neurofibromatosis.

With regard to the abovementioned findings and multiple cervical IDEM lesions [13], we assumed further differential diagnosis for the patient as follows:

1. Plexiform neurofibroma
2. Schwannoma
3. Ganglioneuroma

**Figure 5:**
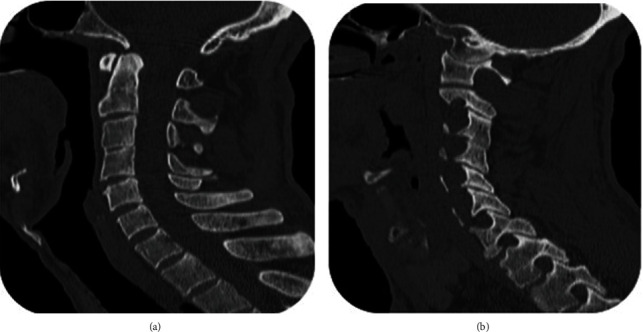
Sagittal spiral CT scan of cervical spine, showing C3-C4 partial fusion (a) and expanded intervertebral foramina mostly at C1-C2 level (b).

Moreover, spiral CT scan of cervical spine revealed C1-C2 intervertebral foramina expansion and C3-C4 fusion, in favor of the Klippel–Feil syndrome (Figure 5).

Having the high probability of NF-1 in mind, the lesions were most probably multiple cervical neurofibroma or schwannoma. On the other hand, slight nerve root involvement signs and symptoms were inconsistent with these diagnoses as ganglioneuroma. Yet to reach a thorough diagnosis and spinal cord decompression, we decided to resect the most spinal cord compressing masses, bilateral C1-C2 and C4-C5, and to investigate them from microscopic point of view. To this aim, the procedure was performed as follows.

After putting the patient at appropriate prone and his neck in neutral fixed position, C1 to C5 vertebrae were made exposed through a midline vertical linear skin incision and paravertebral muscle shave up. Following hemostasis over C2 roots and soft tissue shaving over tumor capsules, bilateral symmetrical C2 enlarged ganglions were exposed and borders released. After performing partial caudal C1 and complete C2 laminectomy, dura was opened and retracted so that bilateral dorsal round masses were seen, displacing spinal cord anteriorly, touching each other near midline with obvious arachnoid planes in between, extending laterally to nerve root sleeves and intervertebral foramina. There was also an arachnoid plane between masses and spinal cord. Both masses had fleshy appearance and were firm but not suckable. Tumors' arachnoid planes were gently dissected and released from spinal cord and dura to the extent that their lateral exiting sites became visible. Then, via the inside-out approach, bilateral C1-C2 dumbbell shape IDEM masses were resected and sent for pathological investigation. Dorsal portion of bilateral C2 roots, which were involved in tumors, were sacrificed. Bilateral C2 root sleeves and dorsal dural opening were repaired secondarily with fascial graft.

Afterwards, laminectomy was performed, complete in C4, and partial in C3 and C5. After dural opening and retraction, bilateral C4-C5 ventrolateral intradural masses were exposed. Both masses were of the same appearance and tenacity as C2 lesions but smaller in size, compressing spinal cord slightly from ventrolateral aspect, pushing it a little dorsal. Gentle arachnoid dissection and release of margins from spinal cord and dura were done bilaterally, and both masses were followed laterally so that exiting site from nerve root sleeves became visible. Both lesions were then resected by sacrificing dorsal portion of involved nerve roots, and samples were sent for pathology. Dural opening and root sleeves were also repaired secondarily using fascial graft. After inserting a hemovac in the surgical site, the wound was tightly closed layer by layer to prevent CSF leakage.

**Figure 6:**
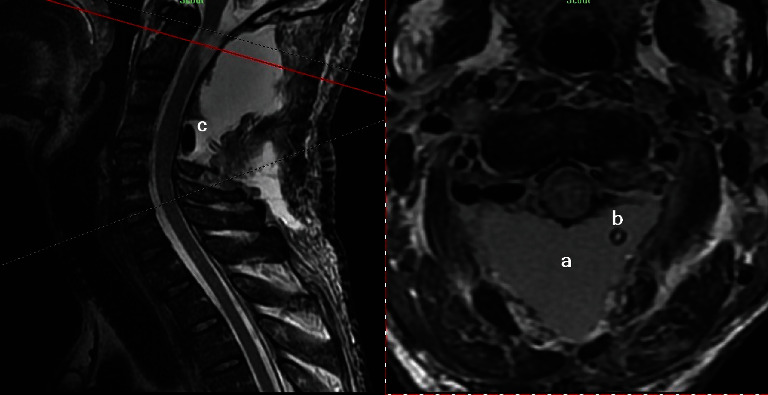
Sagittal-axial cross-view of postoperative T2WI of C1-C2 level showing dorsal CSF collection (a), hemovaccum tube (b), Gelfoam covering dura (c), and no remnant of bilateral C1-C2 GNs.

**Figure 7:**
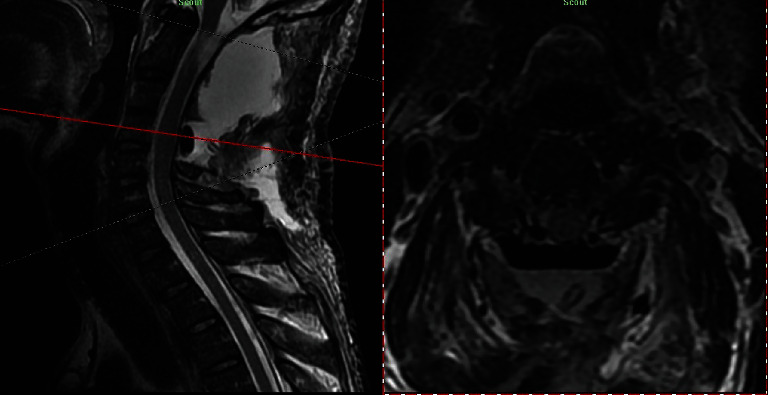
Sagittal-axial cross-view of postoperative T2WI of C4-C5 level showing spinal cord decompression and no remnant of bilateral intradural C4-C5 GNs.

Postoperative early cervical MRI was performed to investigate tumor remnants and spinal cord release reported by radiologist as follows (Figures 6 and 7). “Posterior element of C1-C2-C3 and C4 is not seen, due to previous resection. Evidence of fluid accumulation is seen at posterior aspect of C1-C4 cervical spine, measuring 71 × 27 × 19 mm, which could be due to post op change. Evidence of atrophy of cervical spine is seen at the level of C1-C2, associated with central signal change, which could be due to previous operation and an underlying disease. Prominence of all neural foramen of cervical spine is seen, associated with subcutaneous nodule; all these findings are suggestive of neurofibromatosis. No enhancing lesion is seen after injection of contrast agent.”

**Figure 8:**
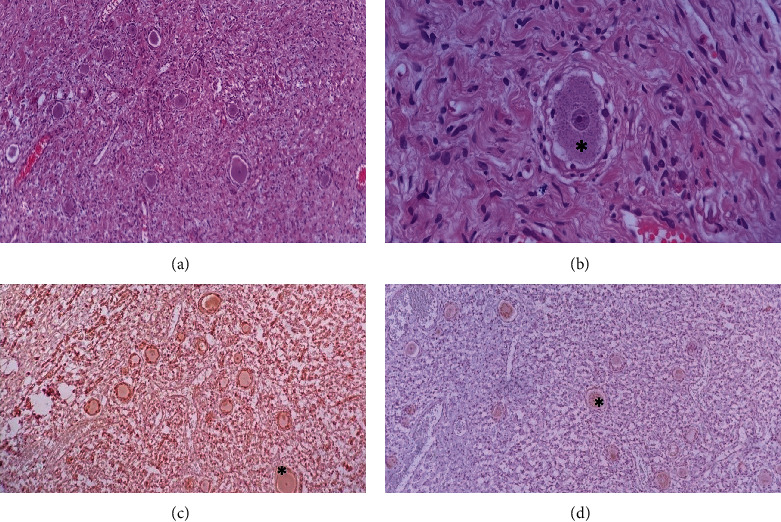
Histopathology of the tumor. (a, b) Histopathological sections of the tumor show bipopulation of the tumor cells, sheet of spindle shape bland looking cells mixed with scattered large spherical ganglion cells (^∗^). Neither necrosis nor mitosis is seen in the tumor (×100, ×400, hematoxylin and eosin). (c) Immunohistochemical stain for S-100 shows diffuse, strong immunopositivity in both neuroma and ganglion cell component (^∗^) (×100). (d) Immunohistochemical stain for chromogranin shows immunostaining in ganglion cell component (^∗^) only (×100).

The patient had CSF leakage through the hemovac; thus, lumbar drainage tube was applied for the patient and kept for a couple of days. After CSF leakage ceased, he was able to walk and to do his self-care; finally, having no fever for 48 hours or signs of active infection, the patient was discharged. Pathologic microscopic investigation of all specimens revealed that encapsulated tumors composed admixture of ganglion, Schwann cells, mature ganglion cells with large nuclei, and prominent nucleolus; Schwann's cells were arranged in fascicles and separated by collagenous stroma. Few eosinophilic granular bodies were seen, and no mitosis and necrosis were identified (Figure 8).

## 3. Discussion

Ganglioneuromas (GNs) are benign slow-growing tumors of ganglion cells with the origin of migrated neural crest cells. They was first described by Loretz in 1870 and in cervical region by De Quervain in 1899 [14]. According to the International Neuroblastoma Pathology Classification (INPC), peripheral neuroblastic tumors (pNT) are classified into 3 major classes, namely, neuroblastomas (NB), ganglioneuroblastomas (GNB with 2 subclasses as nodular (GNB-N) and intermix (GNB-I)), and ganglioneuromas (GN). GNs are a benign form of this spectrum, originating from ganglion cells located in posterior mediastinum, retroperitoneal space, adrenal medulla, spermatic cord, gastrointestinal system, and, rarely, in spinal dorsal ganglion. Its pathologic feature is presence of Schwann cells in a fibrous bed and large cells with interlacing bundle of spindle cells with large vesicular nuclei indicating ganglion cells without atypia, high mitotic index or [7, 15, 16]. In addition, it is known to occur mostly bellow 20 years of age, predominantly in females with more incidences in extraspinal regions. Spinal ganglion involvement has presented in different ages, genders, and presenting patterns [10, 11, 15]. Extraspinal GNs generally present with palpabale masses beside clinical and paraclinical findings of cathecolamin overproduction [14–16]. Spinal GNs are derived from dorsal root ganglia and grow with different patterns. To date, many cases of dumbbell shape cervical/thoracic GNs have been reported. The dumbbell shape growth pattern is due to the ingrowth of tumors into the intervertebral foramina which can become only foraminal or intraspinal (intra- or extradural). The mean age of patients afflicted with this type of tumors ranging from 15 to 72 years, and the main presenting symptoms included mass effect in neighboring structures, mainly, on the spinal cord, and deformities. When becoming an IDEM tumor, spinal GNs are of rare differential diagnosis in comparison to meningiomas, nerve sheath tumors, lipomas, lymphomas, and such. A number of studies have shown diffusion weighted imaging (DWI) to be helpful in differentiating GNs from nerve sheath tumors, with schwannomas having higher apparent diffusion coefficient value than GNs [9]; yet surgical resection—especially when symptomatic—is the most acceptable modality of choice for management of an IDEM tumor including GNs [14, 16–18]. There also seems to be a similar female predominance between spinal dumbbell GNs and extraspinal types. In the matter of association with NF-1, there has also been a significant concordance, but not with a clear genetic basis [1–3, 17, 19]. When it comes to bilateral and symmetrical GNs, it is interesting to notice these points:

- First of all, bilateral symmetrical GNs have been found only in cervical region so far
- They are so extremely rare that only 8 cases have been reported, ours being the 9^th^
- Only men are involved, except one female case
- The age range of affliction is between 15 and 72 years with mean age at 36
- Seven out of 9 cases have occurred in Asia (6 of whom in eastern Asian countries)
- They all present with slowly progressive myelopathy or muscle weakness
- They can occur with or without NF-1, but more significantly, they associate with sporadic NF-1

Here, we discussed the similarities and differences between these bilateral symmetrical cervical GNs, compared to our case. As you can see in Table 1, the first case was reported by Ugarriza et al., (2001) in Spain [20] and the last one by Tan et al. in China [9]. As you notice in Table 1, six of previous cases have been reported in eastern Asia [7–11, 20] (four of which in Japan [8, 10, 11, 20] and two from Europe [4, 21], indicating special geographic or race distribution deserving more attention. All but one case [21] have been male (*M*/*F* = 8/1), in contrast to female predominance in spinal dumbbell shape and extra spinal types of GNs [4, 7], which may be an indicator of different genetic bases and mutations. In all but one case [9], C2 ganglion, assumed as the greatest spinal dorsal root ganglion, has been involved, which may be due to the size and numerous ganglion cells inside. There is also a significant chance of multiplicity in these tumors; 5 out of 9 cases [8–10, 21] including ours, have been multiple—only 1 of which [8] has been multiple symmetrical. It makes our case the second in this aspect, that is a noticeable relation compare to those of asymmetrical dumbbell shape spinal GNs. As mentioned in previous studies [4, 7, 9], there is a strong relationship between NF-1 and bilateral symmetrical GNs, evidenced by 6 out of 9 cases [7–10, 21] having had probable sporadic NF-1 (with only cutaneous manifestations), and one case occurring in a known case of familial NF-1 [20]. Further genetic confirmation studies may be necessary to approve NF-1 diagnosis associated with these tumors, which, in contrast, may reveal entirely different mutations due to different pattern and distribution of these tumors. Intradural extension is an important aspect of these tumors that makes them a differential diagnosis among IDEM tumors. To date, multiple bilateral symmetrical GNs have been found in only two cases [8, 9]:

- In Kyoshima et al.'s case, which was a probable sporadic NF-1, bilateral C1-C2 and C2-C3 masses were grown into dural sac, making a single large ventral mass, extending from the foramen magnum to C4, compressing the upper cervical spinal cord segments, leading to severe myelopathic signs and symptoms [4]
- in Hioki et al.'s case, which did not have any presentations of NF-1, bilateral C1-C2 symmetrical mass was grown into the dural sac and compressed spinal cord

In our case, there are such unique features as follows, probably indicating different or more severe genetic mutations or higher amplifications:

- First, all cervical dorsal root ganglia has been symmetrically and bilaterally involved, causing intervertebral foramina expansion and extra spinal noticeable components in imaging
- Second, at the C1-C2 level, we had dorsal intradural growth and compression in contrast to ventrolateral growth at the C4-C5 level, which was not seen before

## 4. Conclusion

GNs are benign slow-growing tumors consisting of mature ganglion cells and Schwann's cells. They can occur in different parts of the body, more commonly at mediastinum and retroperitoneal space and, rarely, in dumbbell shape spinal forms. Recently reported cases of bilateral symmetrical spinal GNs had different ages, genders, and distribution patterns with strong predominance in men in their 3rd-5th decades of life. They appear to occur only in the cervical spinal region, nearly all involving the C2 dorsal root ganglion.

## Figures and Tables

**Table 1:** Bilateral symmetrical cervical GNs.

Case	Year/place	Age/gender	Involvement	NF-association	Multiple	Intradural extention	Other features
Ugarriza, F. [4]	2001/Spain	53 M	C1-C2	—	—	—	Laminar erosion
Kyoshima, K. [8]	2002/Japan	33 M	C1-C2,C2-C3	Sporadic	+	+	Large IDEM mass
Miyakoshi, N. [10]	2010/Japan	15 M	C1-C2 Lt C3-C4	Sporadic	+	—	—
Bacci, C. [21]	2010/Italy	32 F	C1-C2 Lt C4-C7	Sporadic	+	—	Extraspinal involvement
Ando, K. [20]	2012/Japan	20 M	C1-C2	Familial	—	—	C2-C3,C3-C4 dumbbell Neurofibroma
Hioki, A. [11]	2014/Japan	72 M	C1-C2	—	—	+	—
Chaurasia, B. [7]	2018/Bangladesh	36 M	C1-C2	Sporadic	—	—	—
Tan, C. [9]	2019/China	27 M	C2-C3 C3-C4	Sporadic	+	—	—

## Data Availability

Patient information was accessed through medical records at Shiraz University of Medical Sciences and is unavailable for release due to patient confidentiality.
